# Fractionation of Lignin for Selective Shape Memory Effects at Elevated Temperatures

**DOI:** 10.3390/ma13081940

**Published:** 2020-04-20

**Authors:** Ngoc A. Nguyen, Christopher C. Bowland, Peter V. Bonnesen, Kenneth C. Littrell, Jong K. Keum, Amit K. Naskar

**Affiliations:** 1 Chemical Sciences Division, Oak Ridge National Laboratory, Oak Ridge, TN 37831, USA; bowlandcc@ornl.gov; 2 Center for Nanophase Materials Sciences, Oak Ridge National Laboratory, Oak Ridge, TN 37831, USA; bonnesenpv@ornl.gov (P.V.B.); keumjk@ornl.gov (J.K.K.); 3 Neutron Scattering Division, Oak Ridge National Laboratory, Oak Ridge, TN 37831, USA; littrellkc@ornl.gov

**Keywords:** lignin, shape memory, fractionation, thermomechanical property, intrinsic viscosity, shape fixity, stress recovery

## Abstract

We report a facile approach to control the shape memory effects and thermomechanical characteristics of a lignin-based multiphase polymer. Solvent fractionation of a syringylpropane-rich technical organosolv lignin resulted in selective lignin structures having excellent thermal stability coupled with high stiffness and melt-flow resistance. The fractionated lignins were reacted with rubber in melt-phase to form partially networked elastomer enabling selective programmability of the material shape either at 70 °C, a temperature that is high enough for rubbery matrix materials, or at an extremely high temperature, 150 °C. Utilizing appropriate functionalities in fractionated lignins, tunable shape fixity with high strain and stress recovery, particularly high-stress tolerance were maintained. Detailed studies of lignin structures and chemistries were correlated to molecular rigidity, morphology, and stress relaxation, as well as shape memory effects of the materials. The fractionation of lignin enabled enrichment of specific lignin properties for efficient shape memory effects that broaden the materials’ application window. Electron microscopy, melt-rheology, dynamic mechanical analysis and ultra-small angle neutron scattering were conducted to establish morphology of acrylonitrile butadiene rubber (NBR)-lignin elastomers from solvent fractionated lignins.

## 1. Introduction

Shape memory materials have been reported for various applications in biomedicine [1], packaging [2], actuation [3] (particularly in soft robots [4]), and flexible electronic devices [5]. Materials’ shapes can be programmed and recovered by external stimuli such as light [6,7,8,9], heat [2,5,10], magnetic fields [11], water [12,13,14], pH [3,15], or combination(s) of multiple stimuli [4,16,17,18,19]. One-way shape memory effects using heat to program the material shape is of interest due to its simplicity. The key design aspects for such materials involve utilization of different molecular structures having different thermal responsiveness or contrasting phase change behavior under thermal activation [20]. This mechanism has been tailored to synthesize diverse molecular structures having various shape memory effects. For example, Zarek and colleagues [5] employed the thermal transition temperature of a semi-crystalline polymer, polycaprolactone (PCL) to build three-dimensional (3D) shape memory structures. The 3D-printed structures that were deformed and then fixed at the deformed condition were able to recover their original shapes when exposed to a temperature above the PCL melting point (ca. 43–60 °C), for example 70 °C. Different thermal relaxation of PCL crystalline domains and crosslinked (amorphous) structures resulted in shape programmability and recovery [5].

By using a similar molecular design, we utilized structural characteristics of available lignin (the second most available renewable materials [21]) and an acrylonitrile-butadiene rubber to prepare a highly tough elastomer having excellent shape memory effects [22]. Available crosslinked structure and reactive double bonds of the nitrile rubber were employed to melt-react and entangle with the highly branched lignin structure. Aliphatic and aromatic hydroxyl groups within the lignin structure formed hydrogen (dynamic) bonds with themselves and nitrile groups. Presence of a dual network structure, both chemical and physical crosslinks, resulted in significant enhancement of mechanical stiffness, strain fixity, and glass transition temperature of the materials. A recent report shows a first example of shape memory composites of lignin and nitrile rubber with programmable and switchable electrical conductivity [22]. However, composites of syringylpropane-rich hardwood lignin with nitrile rubber required low programming temperatures. It required fixing the material shape at a low temperature because the lignin-rubber product had a low glassy to rubbery transition temperature. Also, the materials could tolerate only low applicable stress that limited their use as stress/strain sensors.

Lignin has complicated structures containing both aliphatic and aromatic branched backbones with multi-functional groups and linkages such as hydroxyl, carbonyl, β-O-4, methoxy, α-O-alkyl and γ-O-alkyl ethers [23,24,25]. Available reactive groups and 3D network structures of lignin vary its chemical and physical properties and have been investigated widely for developing multifunctional polymeric materials [26,27,28]. Modified lignin functional groups and linkages were used to change its macromolecular structure and corresponding thermomechanical properties. To overcome the temperature and stress limitation of the lignin-nitrile rubber composites, primarily caused by low Tg of the material, we recently reported that thermal annealing of lignin or dynamic shearing of the lignin-nitrile rubber composites for an extended period (1 h) at 180 °C could offer excellent strain recovery and broad applicable stress in these compositions [29]. Thermally unstable lignin ether linkages were activated generating reactive sites to manipulate the crosslink density formed between lignin molecular chains and nitrile rubber network. An increase of over 230% elastic storage modulus and 18 °C glass transition temperature was achievable. The materials unveiled superior elastic work density and a better strain recovery. 

In this work, we apply fractionation of lignin in a selected solvent, acetone, to enrich the lignin functionalities and physical characteristics. Solvent fractionation is a simple method to separate different lignin molecular weights and functional groups based on its solubility in different solvent systems [30,31,32]. Acetone was used in this study because it is a good solvent for syringylpropane-rich hardwood lignin. The majority of this lignin is soluble in acetone; approximately 15 wt.% remains insoluble. The soluble lignin and insoluble lignin exhibit contrasting physical properties that enabled distinct thermomechanical characteristics and shape memory effects of lignin-nitrile rubber composites. We report the detailed studies of the acetone fractionated lignin structures and their correlation to thermal and rheological properties of lignin-nitrile rubber composites that are applied for shape memory effects. 

## 2. Materials and Methods 

Acrylonitrile butadiene rubber (NBR) containing 41 mol % nitrile and organosolv hardwood lignin was provided by Scientific Polymer and Lignol Innovations, Canada, respectively. Acetone and dimethyl sulfoxide (DMSO) were purchased from Fisher Scientific and used without further purification.

As-received lignin (50 g) was mixed overnight with acetone (800 mL) at ambient temperature by a mechanical stirrer. The insoluble lignin (I-lignin) fraction was centrifuged and separated. The soluble lignin fraction (S-lignin) in acetone was collected and separated using a rotary evaporator. Both S-lignin and I-lignin were vacuum-dried for 48 h. The collected lignin was used to melt-react with NBR as reported in our previous study [22]. A selected composition of high lignin content (50 wt.%) in NBR was synthesized using a Brabender Plastic Corder equipped with a 30-cc mixing chamber and high-shear twin roller blades. First, NBR was loaded and pre-mixed for 2 min at 90 rpm and 180 °C. Then lignin was added and mixed for a total of 60 min. After melt-reacting, the samples were collected and stored at ambient temperature for further characterizations. The 50 wt.% NBR-S lignin/I lignin composites were pressed and molded into films between two Teflon sheets at 190 °C for 20 min.

Characterization of fractionated lignins, S-lignin and I-lignin, was accomplished by ^1^H and ^13^C nuclear magnetic resonance (NMR) spectroscopy in DMSO-*d*_6_ at 23 °C on a Varian VNMRS 500 MHz spectrometer (Palo Alto, CA, USA) [23]. The samples for ^13^C NMR contain Chromium (III) acetylacetonate at 0.01 M to shorten the relaxation time for integration purposes. The two lignin samples were also analyzed by Fourier transform infrared spectroscopy (FTIR) using a PerkinElmer Frontier spectroscopy (Waltham, Massachusetts, USA) in an attenuated total reflection (ATR) mode. All measurements were performed at ambient temperature in a wave number range of 500–4000 cm^−1^ using a force gauge of 70 (a.u.) at a speed of 1 cm/s and 4 cm^−1^ resolution. A total of 32 scans/sample were carried out. The background was subtracted and corrected to get signals from the samples only. 

Thermal stability of the samples was investigated by a thermogravimetric analyzer (TGA), Q500-TA instruments (New Castle, Delaware, USA). A sample weight of ca. 15 mg and a ramp rate of 10 °C/min were used. The TGA measurements were carried out in air atmosphere. To remove the residual moisture, the samples were first loaded at ambient temperature then ramped to 100 °C and kept isothermally for 20 min before increasing to 800 °C. The thermal transition characteristics of the samples were investigated by using a differential scanning calorimeter (DSC, Q2000, TA instruments) from −85 °C to 230 °C. A sample weight of ca. 4 mg, a ramp rate of 10 °C/min, and hermetic pans were utilized for the DSC measurements. Data analysis was performed using the Trios Software (Version 5.0.0.44608) provided by TA instruments. 

Morphological characteristics of the samples were studied by scanning electron microscopy (SEM, Hitachi S-4800, Tokyo, Japan). The investigated samples were fractured in liquid nitrogen. The cryo-fractured cross-sections were imaged at different magnifications with an accelerating voltage of 10 kV and a working distance of ca. 10 mm. The samples were also examined by ultra-small angle neutron scattering (USANS) within a *q* range of 0.0001–0.002A^−1^, beamline BL-1A at the Spallation Neutron Source at Oak Ridge National Laboratory.

Rheological characteristics of the samples were studied by using a Discovery Hybrid Rheometer (DHR-3, TA instruments). Flow tests of different solutions of I-lignin and S-lignin in dimethyl sulfoxide (DMSO) were performed to determine their intrinsic viscosity [33]. In these tests, 40 mm parallel Peltier plates were used with precise temperature control at 25 °C. A solvent trap and silicon oil were used to avoid any moisture absorption or solvent evaporation during measuring. Additionally, strain sweeps and frequency sweeps at high temperatures were carried out to investigate the melt behavior of both S-lignin and I-lignin. 25 mm parallel plates were used for all S-lignin measurements due to its very low viscosity. Whereas, I-lignin exhibited very high melt viscosity and was measured with 8 mm parallel plates to avoid overloading the rheometer torque limit. All measurements were determined in the linear regions.

Dynamic mechanical analysis and shape memory characterization of the materials were performed using our previous method [22] with a dynamic mechanical analyzer (Q800-TA instruments). Temperature-dependent elastic modulus was studied at a ramp rate of 3 °C/min and 1 Hz frequency. To determine the shape memory effects, the sample was first ramped to a selected temperature and kept isothermally for 5 min (described specifically in the results and discussion section). After reaching equilibrium, the sample was stretched to an applied strain at a rate of 10%/min. Then the sample was cooled to the fixing temperature and kept isothermally for 10 min. Next, the applied force was set at 0.001 N to release the stress. After fixing, the sample was heated back to the deforming temperature at a ramp rate of 10 °C/min then kept isothermally for 30 min to record strain recovery of the material after deforming and fixing. The process of deforming-fixing-recovery was repeated 3 times.

## 3. Results and Discussion

**Figure 1:**
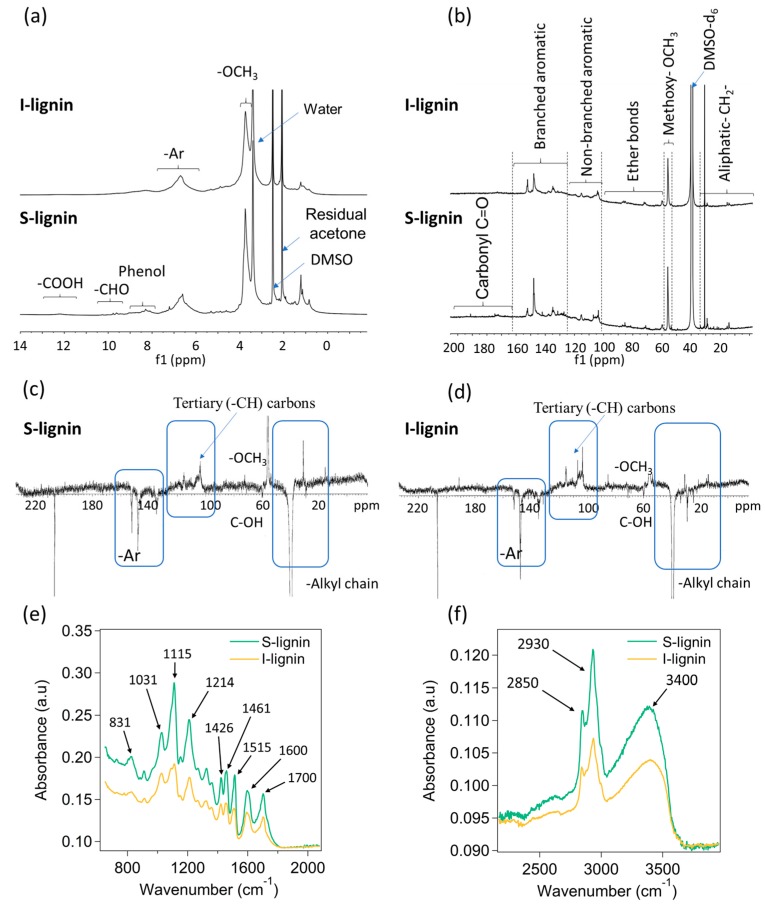
Structural characteristics of I-lignin and S-lignin: (**a**) Proton nuclear magnetic resonance (^1^H-NMR), (**b**) ^13^C NMR, (**c,d**) Attached proton test (APT) ^13^C-NMR, and (**e,f**) FTIR data.

The chemical characteristics of acetone fractionated lignin were carefully investigated by NMR and FTIR. S-lignin and I-lignin showed distinguishable molecular structures evidenced through the dominance of lignin functional groups and representative linkages in S-lignin. The integration of ^1^H NMR peaks was relative to the methoxy region (peak near 3.75 ppm) being set to 3 (±5%). ^1^H NMR results (Figure 1a) demonstrated the presence of aromatic structure domination in S-lignin at ~5.6–7.8 ppm. Peak integration relative to methoxy was 1.64 ± 7% which is higher than the integrated peak (1.29 ± 7%) of I-lignin. In S-lignin the presence of aldehyde and phenol groups as well as -COOH was detected at ~7.8–10.6 ppm (integrated peak 0.57 ± 7%) and ~11–13 ppm (0.07 ± 7%), respectively [34,35,36]. Comparatively, there was no observation of the carboxyl groups in I-lignin. The -CHO and phenol groups determined in I-lignin was only 0.35 ± 7%. Structural characteristics of the fractionated lignin were further examined by ^13^C NMR as shown in Figure 1b [23]. The integral values reported here are relative to the integrated methoxy peak at ~55 ppm region being set to 1. The uncertainty in each integration is around ±5%. The results also exhibited a higher amount of carbonyl group in S-lignin. Integrated peaks from ~168–184 ppm of S-lignin and I-lignin are 0.16 and 0.02, respectively. It was demonstrated that oxygen-containing functional groups such as carbonyl, hydroxyl, methoxy, aldehyde and ether are key factors affecting lignin reactivity [22,29,37,38]. Structure–property relationships of lignin will be further discussed in following sections.

Noticeably, the amount of branched aromatic structure of I-lignin was considerably higher than that of S-lignin. For example, integration of I-lignin’s branched structure was ca. 2.35. In contrast, the corresponding integrated peak of S-lignin was only 1.83. Note that the ether bonds, non-branched and aliphatic structure of both samples are not much different. For example, the non-branched aromatic structure of I-lignin and S-lignin was 1.12 and 0.95, respectively, as measured from the integrated peaks from 98 to 122 ppm (see Figure 1b) [23,24,39]. The presence of high aromatic content in I-lignin could result in a very rigid structure and high melt-viscosity that will be further discussed in the following sections. The integrated peak of ether bonds and aliphatic structure of S-lignin was 0.47 and 0.64, respectively. Comparatively, these areas integrated as 0.57 and 0.57, respectively, for I-lignin. It is noted that the aromatic structure of S-lignin and I-lignin contains dominant tertiary (-CH) carbons (non-branched ones) as evidenced by the chemical shift ~100–130 ppm (See Figure 1c,d, positive peaks). The branched aromatic carbons were indicated by negative peaks in the region of ~130–160 ppm. 

The substantial structural differences of S-lignin and I-lignin were furthered examined by FTIR. Overall, the measured signals obtained from S-lignin were more intense than the signal of I-lignin as shown in Figure 1e,f. Steric hindrance and rigid structure of I-lignin could reduce degree of freedom and restrict the molecular vibration [29,40]. For example, variation of the -C-H aromatic deformation was observed at 831 cm^−1^ [41]. A sharp vibration signal of the C-O deformation in primary alcohol was also determined at 1030 cm^−1^ in the S-lignin data. Noticeably, wide and intense stretching peak for the C-O guaiacyl ring was exhibited at 1214 cm^−1^. Various vibration peaks of aromatic structure were also determined at 1426, 1461, 1515, and 1600 cm^−1^ [41,42,43]. A considerably intense stretching peak for the carbonyl and carboxylic groups in S-lignin was observed at 1700 cm^−1^. Additionally, S-lignin indicated a large amount of C-H stretching in methyl and methylene groups at 2850 and 2930 cm^−1^ (Figure 1f). Contribution of O-H stretching indicates a higher degree of hydrogen bonds in S-lignin as observed in the peak at 3400 cm^−1^.

**Figure 2:**
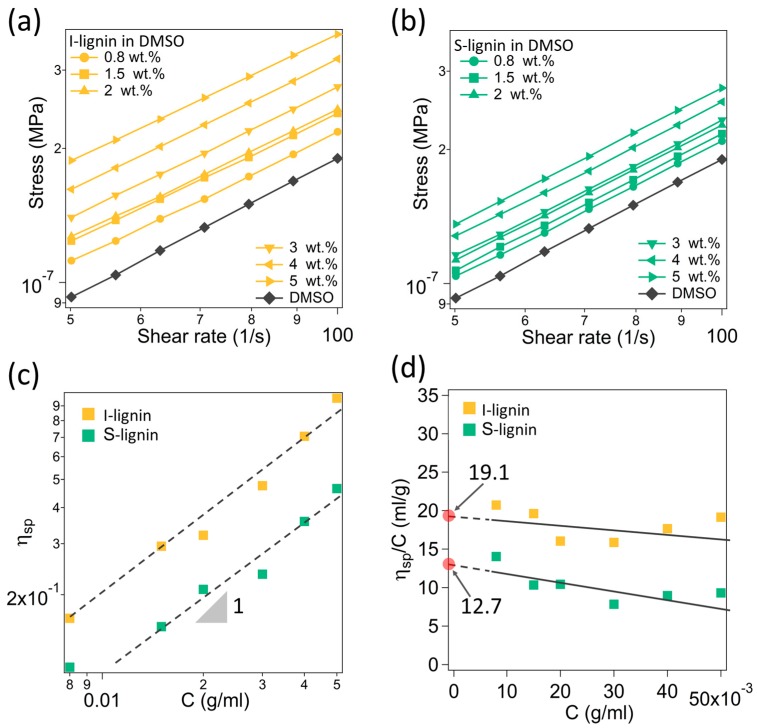
(**a,b**) Stress as a function of shear rate of different solutions of I-lignin and S-lignin in DMSO, respectively; (**c**) Specific viscosity of the studied samples as a function of lignin-DMSO solution concentration (a slope of 1 indicates no molecular interaction); and (**d**) Intrinsic viscosity of insoluble and soluble lignin in DMSO.

In this study, the molecular weight (M_w_) difference of the S-lignin and I-lignin was determined indirectly through their intrinsic viscosity in DMSO. The detailed method was reported elsewhere [33]. The measured rheological data and analysis results showed that I-lignin has a higher molecular weight. Figure 2a,b presents the measured flow characteristics of the lignin-DMSO solutions at different solid weight contents, from 0.8 to 5.0 wt.% in a dilute regime. I-lignin solutions exhibited distinguishably higher shear stress. The measured stress and shear rate data were fitted with the Newtonian model to obtain the viscosity (η) of the solutions. Figure 2c presents the specific viscosity as a function of lignin content (C, g/mL). The specific viscosity (η_sp_) was computed using our previous method [33]. The data was linear fitted revealing a slope of ca. 1 that indicated no molecular interactions [44]. These solutions were truly in the dilute regime. The obtained specific viscosity was employed to determine the intrinsic viscosity of I-lignin and S-lignin (Figure 2d). The extrapolated data of η_sp_/C when C approaches 0 is the intrinsic viscosity of the I-lignin and S-lignin, 19.1 and 12.7, respectively. The molecular weight is proportional to the intrinsic viscosity of the materials [44]. 

**Figure 3:**
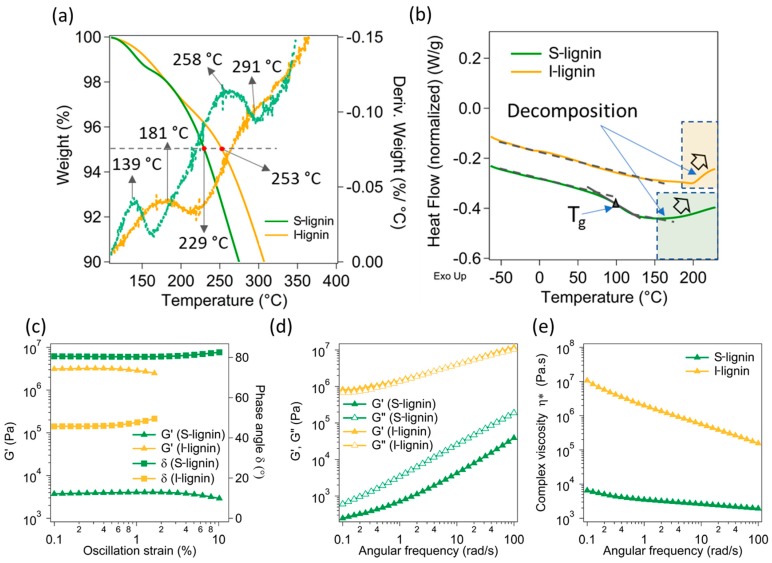
Thermal and rheological characteristics of S-lignin and I-lignin: (**a**) TGA: the solid lines are the weight loss as a function of temperature, the dotted lines are the derivative weight loss; (**b**) DSC; (**c**) Storage modulus (G’) and phase angle (as a function of oscillatory strain; (**d**) Angular frequency dependent storage modulus (G’) and loss modulus (G”); and (**e**) Angular frequency dependent complex viscosity of the studied samples.

Thermal and rheological analyses of both S-lignin and I-lignin were carried out to further understand the effects of their structures and molecular weight. The I-lignin demonstrated a much better thermal stability in comparison to the S-lignin (Figure 3a). It is noted that the first and second degradation temperatures of S-lignin happened at ca. 139 °C and 258 °C. Whereas, I-lignin exhibited the corresponding degradation peaks at 181 °C and 291 °C, approximately 30–40 °C higher. The degradation of lignin occurs in a broad range of temperatures. These degradation peaks were mostly from the remaining moisture release, the cleaved oxygenated functional groups, and small molecular weight fractions [45]. Interestingly, the first and second derivative weight loss (dotted lines) of S-lignin showed sharp and apparent peaks. These behaviors could be due to the presence of a higher low molecular weight fraction and higher oxygenated functional groups of S-lignin as demonstrated in the NMR data (see the previous discussion). For example, 5% weight loss (dashed line, Figure 3a) of S-lignin and I-lignin occurred at 229 °C and 253 °C, respectively. High concentration of aliphatic moieties and low aromatic content in S-lignin also contributed to its low thermal stability.

Additionally, I-lignin exhibited much higher thermal transition temperature in comparison to S-lignin. For example, the glass transition temperature (T_g_) of S-lignin is ca. 100 °C (Figure 3b). Whereas, I-lignin did not indicate any thermal transition at any temperature up to 200 °C. Only an upturn of the heat flow was observed at temperatures above 200 °C. The glass transition of I-lignin and decomposition could happen simultaneously and were not distinguishable. Similarly, the decomposition temperature of S-lignin was also observed at above 150 °C. The DSC data were consistent with the TGA results. We anticipate that the higher thermal transition temperature of I-lignin most likely comes from high molecular rigidity (higher molecular weight and highly aromatic structures).

To have a better understanding of the materials’ thermal characteristics, rheological properties of softened S-lignin and I-lignin were examined and presented in Figure 3c–e). The two fractionated lignin samples revealed contrasting rheological behavior. Specifically, storage modulus (G’) as a function of oscillatory strain of I-lignin (collected at 230 °C) was more than 3 orders of magnitude in comparison to the measured G’ of S-lignin (collected at 150 °C). Note that rheological properties of I-lignin were collected at a much higher temperature than the measurement temperature of S-lignin due to high thermal rigidity of I-lignin. The rheological measurements of I-lignin were conducted at 230 °C to avoid the torque overload. The phase angle of S-lignin was over 80° indicating a liquid behavior (Figure 3c). A similar trend was also determined in the frequency dependent modulus (Figure 3d). I-lignin exhibited an extremely high melt-flow resistance (Figure 3e). The complex viscosity of I-lignin was much higher than that of S-lignin (ca. 2–3 orders of magnitude difference). 

**Figure 4:**
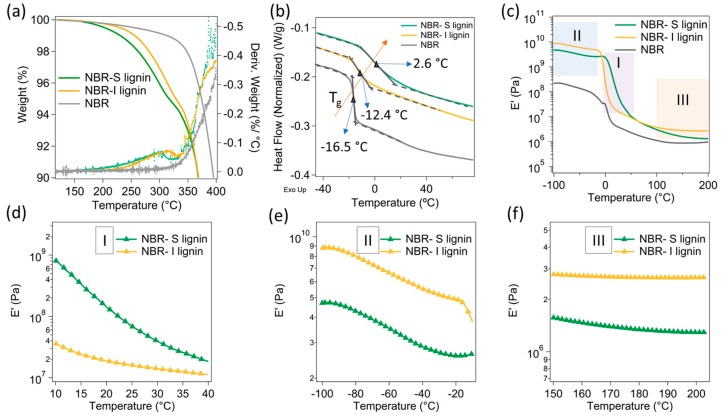
Thermal and dynamic mechanical characteristics of pristine NBR and 50 wt.% NBR–S lignin and I lignin samples: (**a**) TGA: the solid lines are the weight loss as a function of temperature, the dotted lines are the derivative weight loss; (**b**) DSC; (**c**) Temperature dependent modulus (E’); and (**d**–**f**) the corresponding zoomed-in modulus at different temperature ranges.

In this study, the S-lignin and I-lignin were separately melt-reacted with NBR at 50 wt.% loading. The effects of different molecular structures and molecular weight resulted in selective thermal and mechanical characteristics of the lignin/NBR mix. Strong molecular interactions of S-lignin with NBR led to considerable thermal stability enhancement. For example, the NBR-S-lignin composite revealed a similar thermal stability with the NBR-I lignin composite. The first and second degradation peak temperatures of the two samples were not much different, only ca. 10 °C difference (Figure 4a). It was noted that the pristine lignin (S-lignin and I-lignin) revealed a degradation peak temperature difference of ca. 40 °C (Figure 3a). Strong intermolecular interactions of S-lignin with NBR were also demonstrated in the DSC results (Figure 4b). S-lignin resulted in significant increase in T_g_ of the NBR matrix, from −16.5 °C (neat NBR) to 2.6 °C (ca. 19 °C) in the compound (NBR-S-lignin). However, the presence of the I-lignin did not change the T_g_ of the matrix significantly, only ca. 4 °C increase in T_g_ by the addition of I-lignin to the matrix. A lower concentration of oxygen containing functional groups, thermally cleavable linkages, and hydroxyl content in I-lignin (than those in S-lignin) could cause poor interfacial interactions and mild melt-reactivity between NBR and I-lignin [22,29]. As a result, the dynamic mechanical analysis of the materials exhibited high modulus (E’) for NBR-S lignin around ambient temperature (zone I, see Figure 4c,d). Contrastingly, NBR-I-lignin mix indicated high E’ in the zone II and III (Figure 4c) that is mostly due to the presence of I-lignin segments having high molecular rigidity at extremely low temperatures or at elevated temperatures. These distinct differential dynamic mechanical characteristics of NBR-S-lignin and NBR-I-lignin mixes allowed different shape memory effects at ambient temperatures and even at elevated temperatures. 

**Figure 5:**
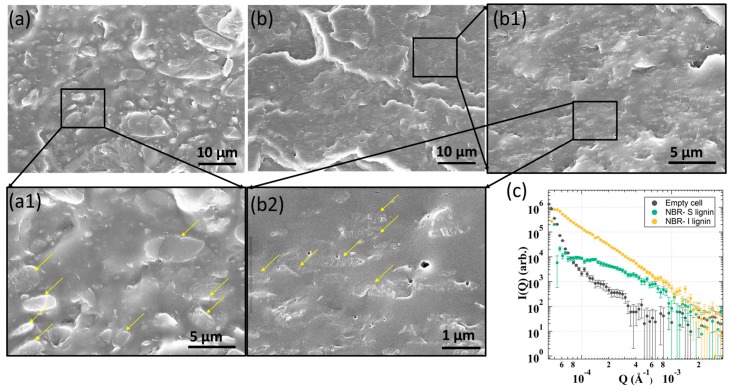
Morphological characteristics of 50 wt.% NBR-S lignin/I lignin samples: (**a,b**) SEM data of 50 wt.% NBR-I lignin and 50 wt.% NBR-S lignin, respectively. (**a1**,**b1**,**b2**) high resolution SEM images of the corresponding samples; (**c**) USANS of NBR-I lignin and NBR-S lignin samples.

Large phase separation and poor interfacial molecular interactions of I-lignin with NBR were examined and shown in Figure 5. A variety of large domains from 5 to 10 µm were observed in NBR-I lignin (Figure 5a(a1)). Yellow arrows indicate a very sharp interface of the lignin phase separated domains in the NBR matrix. The SEM data presented some small voids and defects within the samples that could be due to the lignin degradation during sample processing. Very large micro phase separation of I-lignin was also consistent with the data obtained by USANS as evidenced in Figure 5c. The NBR-I lignin sample revealed a straight line with a power law of ~ −4 after desmearing indicating the presence sharp interface between phase-separated domain and surrounding matrix [46]. In contrast, NBR-S lignin showed very good dispersion of small phase-separated lignin domains within the NBR matrix. Only a small domain size was seen, from 1–2 µm (Figure 5b(b2)). The interface between S-lignin domains and NBR matrix was not very clear suggesting a better interfacial interaction within the composite. Interestingly, the USANS data also agreed very well with the SEM results (Figure 5c). A wide shoulder indicating a characteristic length of ca. 1.5 µm was determined.

**Figure 6:**
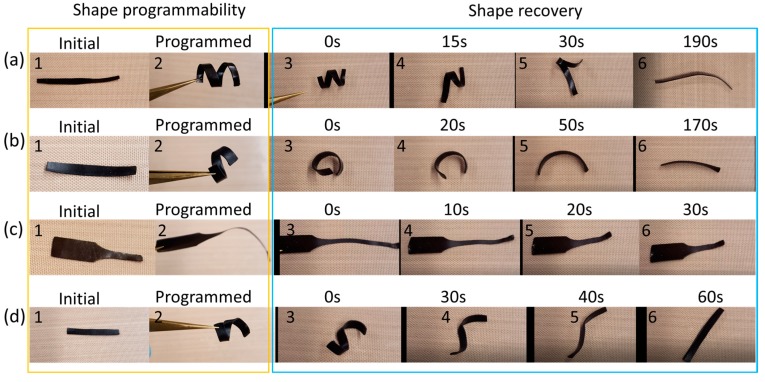
(**a**) Shape programmability (1 and 2) of NBR-S lignin at 70 °C and shape recovery of the sample at 70 °C after fixing. (**b**) Shape programmability (1 and 2) of NBR-I lignin at 70 °C and shape recovery of the sample at 70 °C after fixing. (**c**) Shape programmability (1 and 2) of NBR-S lignin at 150 °C and shape recovery of the sample at 150 °C after fixing. (**d**) Shape programmability (1 and 2) of NBR-I lignin at 150 °C and shape recovery of the sample at 150 °C after fixing.

To demonstrate the shape programmability and shape memory characteristics of NBR- I lignin and NBR-S lignin, selected examples programed at different conditions were performed and shown in Figure 6. The samples were programmed at 70 °C or 150 °C and fixed at ambient temperature, ca. 20 °C. All digital images in Figure 6a–d denoted by 1 and 2 are the initial and programmed shapes of the materials. Figure 6c is an example of the NBR-S lignin having shape programmed by simple stretching indicating that the sample was not yielded even at a high temperature. At ambient temperature, the shape fixity was excellent, and the programmed shape was maintained very well. To recover the initial shape, heat was applied. For example, Figure 6d (images 3–6) showed a process of shape recovery at different time periods after heating the temporarily programmed shape at 150 °C for only 60 s.

**Figure 7:**
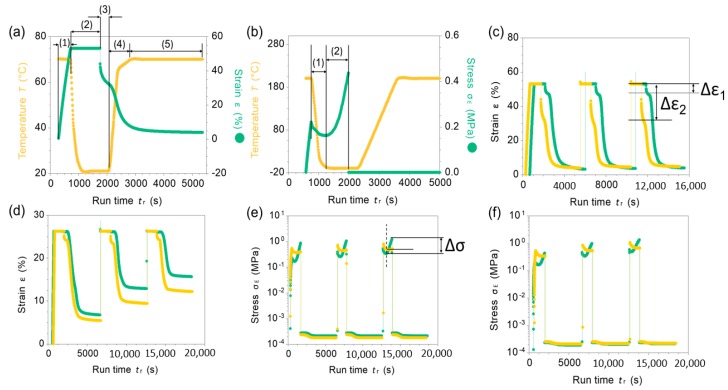
(**a**) An example of a programming process, including (1) material deformation by stretching at a strain rate of 10%/min at 70 °C, (2) fixing process at the selective strain by cooling down to 20 °C, (3) removing the applied stress at the fixing temperature (20 °C), (4) increasing the temperature to 70 °C without the applied stress, (5) keeping isothermally at 70 °C without the applied stress to recover the original dimension. (**b**) An example of a stress profile during the programming process: (1) the applied stress after stretching under cooling process and (2) increasing stress during the fixing process indicating low stress fixity and stability of the studied material. (**c**) Strain recovery after multiple deformation of NBR-S lignin (green profile) and NBR-I lignin (yellow profile) at 70 °C; Δε is the strain loss during fixing process (**d**) Strain recovery after multiple deformation of NBR-S lignin (green) and NBR-I lignin (yellow) at 200 °C. (**e**,**f**) Stress profile of the two studied samples during programming process at two selected temperatures, 150 and 200 °C, respectively (Δσ the stress increase during fixing). NBR-S lignin is green, NBR-I lignin is yellow.

To quantitatively evaluate the shape memory effects of the materials, a detailed study was carried out and presented in Figure 7 (see the experimental section for more details).

In this study, NBR-S lignin demonstrated excellent shape fixity at ambient temperature when programming at a low temperature, 70 °C. In contrast, NBR-I lignin revealed excellent shape recovery and excellent stress stability at an elevated temperature, 150 °C, or even up to 200 °C. Data in Figure 7a,b are examples of a programming process showing shape fixity and stress fixity of NBR-I lignin and NBR-S lignin, respectively. For example, Figure 7a, step 1 is the deformation process, the sample was stretched to 50% strain at 70 °C. After stretching, the sample was kept at that selected strain and the temperature was decreased to 20 °C to fix the temporary shape (step 2). Then, the applied stress was removed (step 3). Note that the strain was dropped in step 3 after removing the applied stress due to a low fixity of NBR-I lignin. Step 4 and 5 is the ramp and isothermal steps to recover the material shape (see the experimental section). An example of stress stability during programming process was given in Figure 7b. Step (1) is the stress profile applied to the NBR-S lignin sample during deforming and fixing processes. However, during isothermal process, step (2), the material exhibited very low stress stability, in which the applied stress increased significantly, from ca. 0.2 MPa to 0.4 MPa. A low stress stability of this material is most likely due to the shrinking of the molecular network in the NBR matrix and low molecular rigidity of S-lignin. However, NBR-S lignin demonstrated very good shape recovery and good strain fixity at ambient temperature. After fixing, the sample still maintained the programmed strain, only ~5% strain loss was measured, see Δε1 (Figure 7c). NBR-I lignin had poor strain fixity at ambient temperature. For example, after fixing, the strain loss of NBR- I lignin was more than 20% (see Δε2, Figure 7c). Low strain fixity of this composite is mostly due to the low glass transition temperature of the material and poor molecular interaction between I-lignin with NBR matrix. The polymeric chains relaxed during fixing. The strain loss resulted from the elastic recovery of the NBR segments. However, the presence of rigid I-lignin resulted in excellent shape recovery and stress fixity at elevated temperatures, such as 150 and 200 °C as demonstrated in Figure 7d–f. We anticipate that the interactions between I-lignin and NBR are more favorable at high temperatures. 

## 4. Conclusions

We have demonstrated that by selecting appropriate lignin through solvent fractionation, the composites of NBR with lignin could be tailored with unique thermal and mechanical characteristics in selected applicable conditions for preferred shape memory effects. The structural characteristics of fractionated lignin were examined and correlated to its thermomechanical properties. Acetone-soluble fractionated lignin had high aliphatic moieties and low branching aromatic structure along with the presence of plentiful functional groups, whereas the insoluble lignin fraction revealed a higher molecular weight in comparison to the acetone soluble lignin fraction. Both fractionated lignin samples exhibited their unique properties that can be utilized for specific shape memory applications. The initial results obtained from acetone fractionation of organosolv hardwood lignin are very promising. Further investigation of selectively manipulated lignin structures by using different solvents combined with multiple-fractionation processes will open a new avenue of lignin valorization for applications in stimuli responsive materials.
